# DNA Damage by Radiopharmaceuticals and Mechanisms of Cellular Repair

**DOI:** 10.3390/pharmaceutics15122761

**Published:** 2023-12-12

**Authors:** Yousef Khazaei Monfared, Pedram Heidari, Samuel J. Klempner, Umar Mahmood, Aparna R. Parikh, Theodore S. Hong, Matthew R. Strickland, Shadi A. Esfahani

**Affiliations:** 1 Division of Nuclear Medicine and Molecular Imaging, Department of Radiology, Massachusetts General Hospital, Harvard Medical School, Boston, MA 02114, USA; ykhazaeimonfared@mgh.harvard.edu (Y.K.M.); heidari.pedram@mgh.harvard.edu (P.H.); umahmood@mgh.harvard.edu (U.M.); 2 Division of Hematology-Oncology, Department of Medicine, Mass General Cancer Center, Massachusetts General Hospital, Harvard Medical School, Boston, MA 02114, USA; sklempner@mgb.org (S.J.K.); aparna.parikh@mgh.harvard.edu (A.R.P.); mstrickland1@mgh.harvard.edu (M.R.S.); 3 Department of Radiation Oncology, Massachusetts General Hospital, Harvard Medical School, Boston, MA 02114, USA; tshong1@mgh.harvard.edu

**Keywords:** DNA damage, DNA repair, radiopharmaceuticals, radioisotope, cancer

## Abstract

DNA is an organic molecule that is highly vulnerable to chemical alterations and breaks caused by both internal and external factors. Cells possess complex and advanced mechanisms, including DNA repair, damage tolerance, cell cycle checkpoints, and cell death pathways, which together minimize the potentially harmful effects of DNA damage. However, in cancer cells, the normal DNA damage tolerance and response processes are disrupted or deregulated. This results in increased mutagenesis and genomic instability within the cancer cells, a known driver of cancer progression and therapeutic resistance. On the other hand, the inherent instability of the genome in rapidly dividing cancer cells can be exploited as a tool to kill by imposing DNA damage with radiopharmaceuticals. As the field of targeted radiopharmaceutical therapy (RPT) is rapidly growing in oncology, it is crucial to have a deep understanding of the impact of systemic radiation delivery by radiopharmaceuticals on the DNA of tumors and healthy tissues. The distribution and activation of DNA damage and repair pathways caused by RPT can be different based on the characteristics of the radioisotope and molecular target. Here we provide a comprehensive discussion of the biological effects of RPTs, with the main focus on the role of varying radioisotopes in inducing direct and indirect DNA damage and activating DNA repair pathways.

## 1. Introduction

**Figure 1:**
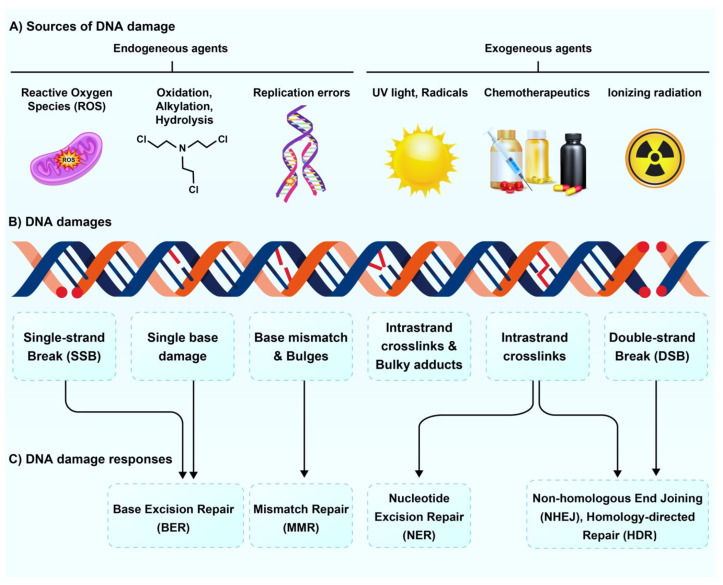
Schematic illustration of various factors that can induce DNA damage. (**A**) The upper panel demonstrates examples of endogenous and exogenous damaging factors, and (**B**) the middle panel shows the resultant DNA damage mechanisms caused by these factors. (**C**) The lower panel provides a list of the triggered DNA repair pathways in response to the DNA damage.

DNA is a vital repository of genetic information necessary for the development of organisms and the sustenance of life [1,2]. As the fundamental unit of inheritance, DNA is an organic molecule that exhibits relatively high stability compared to other biological compounds in the body. However, it is highly susceptible to chemical alterations caused by both internal and external factors. Additionally, errors can occur during DNA replication and repair, leading to the accumulation of potentially harmful mutations within cells [3]. Biologic processes in normal cellular homeostasis subject human cells to over 10,000 instances of DNA damage, which may trigger the onset of cancer, various human diseases, and the aging process [4]. DNA damage mechanisms can be mainly categorized as endogenous and exogenous [2,5]. Endogenous DNA damage occurs naturally within the cells as part of the normal cellular processes, for instance, by aberrant DNA methylation or malfunction of topoisomerases [6]. Other common causes of endogenous DNA damage include replication errors, DNA base mismatch, spontaneous base deamination, and oxidative DNA damage by reactive oxygen species (ROS) [7,8]. Exogenous DNA damage is caused by external factors, including environmental, physical, and chemical factors such as ultraviolet and ionizing radiation, alkylating agents, and crosslinking agents [2,5] (Figure 1A). These damages can lead to a DNA single-strand break (SSB) or double-strand break (DSB), which refer to the breakage of one of the DNA strands while the other strand remains intact and the breakage of both DNA strands, respectively [9] (Figure 1B).

Cells possess complex and advanced mechanisms, including DNA repair, damage tolerance, cell cycle checkpoints, and cell death pathways, which together minimize the harmful effects of DNA damage. When cells encounter DNA damage, they activate strong DNA damage response (DDR) pathways, which provide an adequate timeframe for specific pathways to physically repair the damage, depending on the nature of the impaired component. Cells employ at least five major DDR pathways: Base excision repair (BER), nucleotide excision repair (NER), mismatch repair (MMR), homologous recombination (HR), and non-homologous end joining (NHEJ) [5,10,11] (Figure 1C). In many cancer types, the fidelity of DNA damage tolerance and response becomes disrupted or deregulated. This disruption contributes to increased mutagenesis and genomic instability within the cancer cells, which leads to disease progression [3,12,13]. This inherent instability of the genome in rapidly dividing tumors can be exploited via multiple external mechanisms, such as chemotherapeutics, external beam radiation therapy (EBRT), or systemic radiopharmaceutical delivery [3,14]. Radiopharmaceutical therapy (RPT) is an emerging approach in nuclear oncology. Using molecularly targeted constructs linked to a radioisotope, radiopharmaceuticals specifically accumulate in the tumors and induce tumor cell nuclear damage and cell death that are reflected clinically by the imaging response [15,16,17].

The potential for RPT has been exemplified by the somatostatin receptor (SSTR) and prostate-specific membrane antigen (PSMA) targeted radiopharmaceuticals, which have received Food and Drug Administration (FDA) approval for the treatment of patients with neuroendocrine and prostate tumors, respectively [16,18]. As the field of targeted RPT is growing, it is crucial to have a deep understanding of the impact of these agents on tumors while reducing off-target damage. The distribution and nature of DNA damage and repair pathways caused by systemic radiation delivery can be different based on the type of radioisotopes used in addition to the characteristics of the specifically targeted molecules in the tumors [5,13]. This article provides a comprehensive review of the impact of different radioisotopes on the induction of DNA damage and repair pathways while highlighting knowledge gaps in the field that will be critical to realizing the full potential of RPT in solid tumors.

## 2. Effect of Radioisotopes on DNA Damage and Repair Pathways

Various methods have been used in the review of the literature to assess the biomarkers and mechanisms of DNA damage by radionuclides. The most commonly employed techniques are comet assay (single cell gel electrophoresis), ELISA (enzyme-linked immunosorbent assay), immunoblotting (Western blot), immunohistochemistry (IHC), flow cytometry, and fluorescence microscopy [19,20,21,22,23,24,25].

### 2.1. Emission Properties of Radioisotopes Used for Radiopharmaceutical Therapy

**Figure 2:**
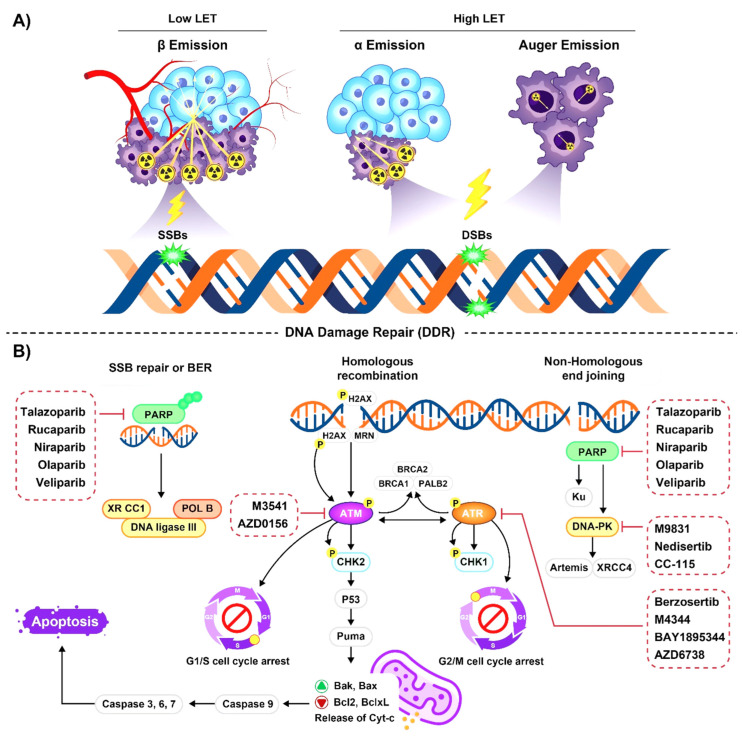
(**A**) Visualization of DNA damage induction patterns of beta-emitters, alpha-emitters, and auger electron emitters exhibits distinct patterns of DNA damage induction based on their penetration range and complexity of DNA damage. Beta-emitters induce isolated lesions over a longer range, alpha-emitters induce more complex damage in a localized area, and auger electrons generate high-density damage in close proximity to the DNA molecule. (**B**) The general overview of DNA damage repair (DDR) pathway initiation by sensor proteins that detect DNA damage, followed by activation of transducer proteins and subsequent phosphorylation of effector proteins. The activation of cell cycle checkpoints and recruitment of DNA repair factors are key components of the DDR, ensuring accurate repair of DNA lesions and maintenance of genomic stability. The DDR inhibitors are shown in red boxes. ATM: ataxia-telangiectasia mutated, ATR: ataxia-telangiectasia rad3-related protein, DNAPKs: DNA-dependent protein kinase, PARP: poly adenosine diphosphate-ribose polymerase, CHK1/2: checkpoint kinase1/2, Bax: B cell lymphoma-associated X, Bcl-2: B cell lymphoma-2, Cyt-c: cytochrome c, BER: base excision repair, SSB: single-strand break, DSB: double-strand break, LET: linear energy transfer.

The radioisotopes used for RPT emit beta (β) or alpha (α) particles [26,27,28,29], or Auger electrons (AE) [30,31]. A summary of the major radioisotope types and their specific characterizations is shown in Table 1 and Supporting Information, Table S1. As the energy travels through the tissues, it gets deposited within the cells, and [17] induces DNA SSB and/or DSBs [15,32] (Figure 2A). To ensure optimal destruction of the targeted cells while minimizing ionization interactions with healthy cells, it is crucial to consider multiple factors such as the particle energy, emission range, and linear energy transfer (LET) in addition to the physical or biochemical characteristics (phenotype), the dimensions or physical extent (size), and location of the target cells within the tumors [5,12,14,15,16,17].

### 2.2. Radioisotopes Emitting High-LET Particles

#### 2.2.1. Alpha-Particle Emitters

The utilization of α-particle emitters as targeted therapeutics has gained substantial interest. Given their high energy, ranging from 4 to 8 MeV, and short emission range in body tissues, a targeted radiopharmaceutical radiolabeled with an α-emitter can lead to high-energy deposition within the tumor while minimizing radiation to the surrounding non-diseased host tissues [39,40,41]. Radium-223 dichloride (^223^RaCl_2_, Xofigo) is the first approved targeted α-RPT by both the FDA and the European Medicines Agency (EMA) for the treatment of patients with metastatic castration-resistant prostate cancer (mCRPC) with bone metastases [42,43,44,45]. Patients treated with ^223^RaCl_2_ have shown significantly improved overall survival (OS) and delayed symptoms from osseous metastases [46,47]. Over the last few years, several RPTs with other α-emitting radioisotopes have been under investigation in clinical trials. These isotopes include astatine-211 (^211^At), bismuth-212 (^212^Bi), bismuth-213 (^213^Bi), actinium-225 (^225^Ac), lead-212(^212^Pb), americium-241 (^241^Am), and plutonium-238 (^238^Pu) [48,49,50].

In a pioneering study, Narayanan et al. demonstrated that ^238^Pu, induced both direct and indirect DNA damage in normal human lung fibroblasts by the formation of hydroxyl radicals and the generation of reactive oxygen species (ROS), respectively [51]. The ^223^Ra inhibits the growth of osseous metastases mainly by inducing DNA DSB in mouse models that received ^223^RaCl_2_ [52]. Researchers discovered that the decay of ^223^RaCl_2_ results in the formation of track-like patterns of DDR proteins, mainly 53BP1 (known as α-tracks) and gamma-H2AX (γ-H2AX) foci, in both peripheral blood mononuclear cells and tumor cells in patients with prostate cancer (PCa) treated with ^223^RaCl_2_ [53,54]. This process leads to the generation of numerous clustered DSBs in PCa cells and activates NHEJ repair pathways [54]. Furthermore, ^223^Ra’s effectiveness in inducing apoptosis and inhibiting the growth of prostate cancer cells by augmenting the caspase 3/7 pathway implies that it might trigger a cascade of events within these cells, leading to their programmed death [54]. In contrast, some in vitro studies have suggested that the formation of γ-H2AX foci does not correlate with the sensitivity of cancer cells to α-radiation [55,56]. In a study by Schumann et al. there was no significant difference in the production of DNA damage caused by radiation when human blood leukocytes were exposed to two radium isotopes (^223^Ra and ^224^Ra) ex vivo [57,58]. The similarity in the decay characteristics of the two Ra isotopes may be attributed to the absence of disparity in their ability to induce radiation-induced DNA damage. Therefore, it may be concluded that the DNA damage elicited by the two Ra isotopes is comparable for the same absorbed doses. This observation underscores the importance of considering the potential risks associated with exposure to these isotopes in a clinical setting, as it suggests that similar precautions and monitoring may be warranted when dealing with either of them.

In an in vivo study, Yong et al. revealed that ^212^Pb-TCMC-trastuzumab, which specifically targets human epidermal growth factor receptor 2 (HER2), in mice carrying intraperitoneal xenografts of human colon cancer, induced apoptosis, DNA DSBs, and suppressed DNA synthesis 24 h after treatment initiation. Furthermore, the expression of the Rad51 protein was observed to be decreased, suggesting a delay in the repair via the HR pathway of DNA DSB compared to the control groups [59].

In addition, this group has shown that administration of ^212^Pb-TCMC-trastuzumab following treatment with gemcitabine resulted in an increased rate of apoptosis, specifically in S-phase-arrested tumors, and induced DNA-DSB, which led to interference with the HR mechanism by the downregulation of Rad51, inhibition of Chk1 phosphorylation, chromatin modification, and disruption of the cell cycle [60]. In a xenograft colon cancer model, ^212^Pb-TCMC-trastuzumab led to reduced cell proliferation by inducing G2/M arrest, blockage of DDS repair, and apoptosis. Additionally, this radio-immunotherapeutic approach upregulated genes linked to DNA damage (DSB and SSBs) without affecting DSB repair genes. It has also led to an increase in stressful growth arrest conditions by inducing cell death-associated genes. This was evidenced by the upregulated expression of genes such as the growth arrest and DNA damage-45 (GADD45) family, as well as p73, a tumor suppressor gene belonging to the p53 family of transcription factors, which are involved in the regulation of apoptotic processes [61]. Furthermore, it was observed that the induction of cell death in gastric cancer cells by ^213^Bi-labeled d9Mab, targeting d9-E-cadherin, was characterized by G2 arrest and the up-regulation of genes known to inhibit apoptosis while promoting necrosis and mitotic catastrophe [62]. ^213^Bi-labeled anti-CD20 and anti-CD45 have demonstrated treatment efficacy in radio- and chemo-resistant non-Hodgkin lymphoma and leukemia by activating caspase 2, 3, 8, and 9 apoptotic pathways [63,64]. The comparative study emphasized that the α-emitting, ^213^Bi labeled to anti-CD33 monoclonal antibody (mAb) WM53, killed the acute myeloid leukemia (AML) cells through inhibiting DNA synthesis, while the β-emitting, samarium-153 (^153^Sm), was not effective when using the tritium-labeled thymidine method [65].

Dimethyl sulfoxide (DMSO) has been widely used as a radical scavenger to prevent indirect radiation-induced DNA damage by neutralizing ROS [66,67]. The Harms-Ringdahl group demonstrated that the error-prone NHEJ pathway was significantly impaired by the indirect effect of α-radiation (with high-LET) compared to other repair pathways investigated, while the indirect effect of gamma (γ)-rays (with low LET) significantly impaired both NHEJ and HRR pathways [68]. In a comparative study, α-emitter, ^241^Am, demonstrated a greater capability for causing severe DNA damage, as shown by increased chromosome rearrangements and cell death, compared to γ-ray, despite inducing a similar number of γ-H2AX foci [69]. Different studies revealed another important class of DNA damage, which is called complex DNA damage (CDD), which refers to the occurrence of two or more DNA lesions in proximity [70,71]. The difficulty in repairing CDD was observed in in vitro synthetic oligonucleotide substrates and in bacterial, yeast, and mammalian cells using plasmid reporter systems [70,72,73]. Based on mathematical modeling, Monte Carlo track structure simulations [74], it has been estimated that the occurrence of CDD rises from around 30% for low-LET radiation to approximately 90% for the highest-LET, α-emitters [75,76,77]. A study conducted by Carter et al. demonstrated that the presence of histone H2B ubiquitylated (H2Bub) on lysine 120 was selectively triggered several hours following exposure to high-LET, α-particles, and protons, but not by low-LET protons or X-rays/γ radiation. This increase in H2Bub levels was associated with elevated levels of CDD and ultimately led to decreased cell survival [78].

Based on the available evidence, α-emitting radioisotopes with high-energy and charged features can directly interact with the DNA molecule and cause the formation of ionized atoms or molecules. This ionization can mainly lead to irreversible and complex DSBs within the DNA [79,80] (Table 2). In response to these α-particle-induced DSBs, cells employ two primary pathways for DNA repair: NHEJ, which works throughout the cell cycle and the G1 phase, and HRR, which primarily functions during the S and G2 phases of the cell cycle [2,12,76,81]. The key proteins in these pathways include ataxia-telangiectasia mutated (ATM), ataxia-telangiectasia rad3-related (ATR), and DNA-dependent protein kinase (DNAPKs) [46,82,83]. On the other hand, one of the immediate effects of DSB is the addition of a phosphate group to Ser139 of the minor histone H2 variant, H2AX, within the large DNA regions near the DSB, which is called γ-H2AX [84]. DSBs also serve as a magnet for the damage sensor called p53-binding protein 1 (53BP1), which is attracted to the adjacent chromatin (Figure 2B). The presence of γ-H2AX or 53BP1 results in the formation of nuclear foci under microscopy [85].

Consequently, the consistent observation of γ-H2AX and 53BP1 foci, known as DNA damage tracks, has been a common finding in studies involving α-emitting radioisotopes [57,58,86,87]. While α-emitters primarily cause direct DNA damage, they can also induce some levels of indirect DNA damage through free radicals [88]. However, the distinction in long-term clinical impact between direct and indirect DNA damage remains unclear and requires further investigation in future studies.

#### 2.2.2. Auger Emitters

Auger electrons (AEs) are extremely low-energy electrons emitted during the decay process of radioisotopes that undergo electron capture [89]. Radioisotopes such as bromine-77 (^77^Br), indium-111 (^111^In), iodine-123 (^123^I), iodine-125 (^125^I), and gallium-67 (^67^Ga) are the most commonly used AE emitters [90,91]. Deposit their energy within nanometer to micrometer distances; these electrons have high LET. The high LET of AE makes them particularly effective in inducing lethal damage to cancer cells [92]. Successful utilization of AE emitters for RPT requires specific targeting of molecules inside the tumor cells, specifically within the nucleus, to allow maximal DNA damage [93,94]. Numerous studies have consistently shown that AE radiation primarily causes direct DNA damage rather than ROS-mediated indirect damage [95,96,97,98,99]. The pioneering work by Kassis and Adelstein groups used ^125^I- or ^123^I-5-iodo-2-deoxyuridine, a compound that gets incorporated into DNA during cell division [100,101], which showed profound cytotoxicity by inducing DSBs and chromosomal aberrations [102]. Additionally, the ^125^I radioisotope decay resulted in DNA DSBs within a 10-base pair region surrounding the site of decay [95].

Various strategies, including the utilization of specific targeting agents or nuclear localization sequences (NLS), have been employed to augment the delivery of AE emitters to the DNA of cancer cells [103]. In this context, numerous studies have substantiated the efficacy of NLS peptides in facilitating the delivery of ^111^In-labeled anti-γH2AX and anti-CD33 (HuM195) mAbs into the nuclei of breast and AML cancer cells. This approach induces lethal DNA damage by increasing γH2AX foci in both cancer models [103,104,105]. Furthermore, it has been observed that the application of ^111^In conjugated with antibodies results in an increased magnitude of DNA damage, characterized by a higher number of γH2AX foci in breast cancer [106,107]. Moreover, the targeting of carcinoembryonic antigen (CEA)-expressing cancer cells using ^125^I-labeled anti-CEA mAbs leads to heightened DNA damage, as evidenced by an increase in γ-H2AX foci formation, independently of apoptosis and the p53 pathways [108,109]. The augmented cytotoxicity observed with ^111^In-NLS-trastuzumab, an anti-HER2 RPT, is substantiated by the enhanced formation of γ-H2AX foci. This indicates the successful delivery of AE radiation to the nucleus and the induction of significant DNA DSBs in breast cancer cells compared to ^111^In-trastuzumab or non-radiolabeled trastuzumab [106]. In contrast, another study involving ^111^In-DOTA-trastuzumab demonstrated no induction of cell death or DNA breakage, suggesting that the treatment may not have effectively penetrated the nucleus of target cells—a crucial step in demonstrating AE-induced DNA damage at the cellular level [110].

Another potential strategy for delivering AE into the nucleus involves the use of radiolabeled fluorescent dyes [111,112]. In a study, DAPI (4,6-diamidino-2-phenylindole) [113], a fluorescent probe that specifically interacts with DNA, was labeled with ^99m^Tc using a HYNIC (6-hydrazinonicotinic) linker, resulting in ^99m^Tc-HYNIC-DAPI. This compound exhibited more pronounced DNA damage in plasmids, including both SSBs and DSBs, when compared to unbound ^99m^Tc-pertechnetate [99]. Additionally, the observed cytotoxicity associated with a DNA-binding Hoechst-tagged radioiodinated BODIPY derivative, ^125^I-BH, was attributed to the induction of DNA DSBs in HeLa cells [114].

To summarize, it is important to highlight that the induction of direct or indirect DNA damage resulting from the short-range and high-energy deposition of AE depends on various factors, including the energy of the AE, its proximity to the DNA molecule, and the surrounding cellular environment [90,115,116,117] (Table 2). Consequently, the selective delivery of AE to specific cellular and nuclear compartments holds promise for enhancing the effectiveness of radiotherapeutic interventions while minimizing harm to healthy tissues.

### 2.3. Radioisotopes Emitting Low-LET Particles

#### Beta Particle Emitters

Radiopharmaceuticals with β-emitting radioisotopes are very commonly used for the treatment of cancer in both clinical and preclinical models. These isotopes include lutetium-177 (^177^Lu), holmium-166 (^166^Ho), rhenium-168 (^168^Re), rhenium-188 (^188^Re), copper-67 (^67^Cu), promethium-149 (^149^Pm), gold-199 (^199^Au), samarium-153 (^153^Sm), rhodium-105 (^105^Rh), strontium-89 (^89^Sr), yttrium-90 (^90^Y), and iodine-131 (^131^I) [118,119,120,121]. The toxicity of β-emitters in mammalian cells is predominantly attributed to indirect DNA damage resulting from the generation of ROS and subsequent oxidative stress [121,122]. These processes primarily result in the formation of SSBs, with fewer DSBs observed in the exposed cells [13,15]. Studies revealed that β-emitters such as ^177^Lu and ^90^Y exert their therapeutic effects primarily through the generation of SSBs. While tumor cells possess repair mechanisms for SSBs, the accumulation of these breaks and the cumulative DNA damage can overwhelm these repair mechanisms, ultimately leading to cell death and tumor shrinkage [123]. However, in the case of treatment with ^131^I for patients with differentiated thyroid carcinoma [124,125], ^177^Lu-DOTATATE for patients with neuroendocrine tumors [126], and ^177^Lu-PSMA for patients with PCa [127], a time- and dose-dependent induction of DSB has been observed, as evidenced by the formation of γ-H2AX and 53BP1 nuclear foci. Octavia et al. showed the presence of nuclear foci in the peripheral blood lymphocytes of patients with thyroid cancer as long as 24 months after treatment with ^131^I [128]. The effect of ^177^Lu, the most commonly used β-emitter, on inducing cell death mechanisms has been extensively investigated [129,130,131,132,133]. ^177^Lu-octreotate increases the activation of poly(ADP-ribose) polymerase-1 (PARP1), a DNA repair enzyme, after internalization in SSTR2-expressing and SSTR5 neuroendocrine tumor cells [131]. The γ-H2AX foci, induced by ^177^Lu-DOTATOC, have been reported as predictors of response to treatment in SSTR-expressing neuroendocrine tumor cells in both in vitro and in vivo models [134,135]. A preclinical study also revealed a better treatment response characterized by reduced tumor growth and increased median survival in a neuroendocrine mouse model treated with ^177^Lu-DOTA-JR11, an SSTR antagonist, compared to ^177^Lu-DOTA-octreotate, an SSTR agonist, by inducing two times more 53BP1 foci formation [132]. Furthermore, the formation of γ-H2AX can serve as an in vivo marker for evaluating the toxicity of normal tissue after long-term internal irradiation with ^177^Lu-DOTA-octreotate [136]. O’Neill et al. provided evidence that the uptake of the single-photon emission computed tomography (SPECT) probe, ^111^In-anti-γH2AX-TAT, increased in the tumor cells responding to treatment with ^177^Lu-DOTATATE. This increase in probe uptake was further confirmed by histology [135]. However, another study demonstrated that ^177^Lu-DOTATATE was not significantly effective in inducing DNA DSBs, as assessed by the levels of γH2AX/pATM, in six human cancer cell lines expressing SSTRs, unlike the substantial effect observed with EBRT [137]. In addition, another study showed that the administration of ^177^Lu-trastuzumab induced cell death by causing DNA DSBs, activating caspase-3-mediated apoptosis, interfering with the expression of DNA-PK, and downregulating various genes involved in DDR, including BRCA1, EXO1, FEN1, MSH2, NBN, PRKDC, and RAD51 [138]. When DNA damage remains unrepaired, it can lead to cell death through either mitotic catastrophe or apoptosis [130,139,140]. Activation of apoptosis in cancer cells by β-radiation is mediated by the p53 signaling pathway [121,129] as well as upregulation of CD95 ligand (FasL) and CD95 receptor (FasR) expression [141,142]. Notably, studies have demonstrated that ^177^Lu induces apoptotic cell death by activating the apoptotic signaling pathway through the downregulation of anti-apoptotic B-cell lymphoma 2 (bcl-2) family genes in human histiocytic lymphoma [139]. In osteosarcoma cell lines, ^177^Lu conjugated to ethylenediamine tetramethylene phosphonic (EDTMP) and 1,4,7,10-tetraazacyclododecane-1,4,7,10-tetramethylene phosphonic acid (DOTMP) induces G2/M phase cell cycle arrest [143] and DNA fragmentation-associated apoptotic cell death. This is achieved by downregulating bcl-2 and cleavage of the PARP protein, which serves as a substrate for active caspase-3 during cell death [144]. In gastrointestinal cancers, ^177^Lu-labeled minigastrin analog therapy induces the DDR, dependent on the presence of functional p53 [145]. On the other hand, ^131^I exhibits a G2-M phase arrest and apoptosis through the activation of initiator caspases-2, -8, -9, and effector caspase-3, along with the PARP cleavage in HeLa Hep2 cells [146]. In human thyrocyte cells, ^131^I induces apoptosis by downregulating bcl-2 and upregulating Fas gene expression. Additionally, ^131^I exposure increases the expression of GADD45, leading to G2/M phase arrest through a p53-independent pathway [138]. Moreover, a study revealed that ^131^I increases cell cytotoxicity and induces apoptosis in Burkitt’s lymphoma, epidermoid carcinoma, and breast cancer cells when compared to an equivalent dose of γ-radiation. This differential effect was associated with variations in the expression of DNA repair genes RAD51 and P21 [137]. Furthermore, ^89^Sr radiation induces G2-M phase arrest and apoptosis by regulating the p53 and bcl-2 genes in various cancer cells [130,139].

It is essential to acknowledge that when β-emitters traverse biological tissue, the majority of DNA damage predominantly arises indirectly via the generation of free radicals, such as ROS, and ensuing chemical reactions. These can potentially induce early or late apoptosis or mutations that result in genomic instability [147]. Some of the major biomarkers that change during this process are FasL, FasR [142,148,149], bcl-2 [150,151], bax [151,152], and PARP, which play a critical role in the transient detection and repair of SSBs in DNA through the long patch BER pathway [131], and caspase-3 [148]. Although indirect damage appears to be the main mechanism, β-particles can also induce direct DNA damage through physical interactions. In certain instances, the β-emitter itself can collide with the DNA, resulting in the displacement of electrons and subsequent ionization and breakage of chemical bonds within the DNA, which can manifest as SSBs and reversible DSBs [140,141]. Two important pathways involved in repairing DNA damage caused by β-emitters are BER and NHEJ [15] (Table 2). Furthermore, β-radiation has been observed to enhance the formation of γ-H2AX and 53BP1 nuclear foci in various cancers [115,124,125].

**Table 2:** The role of different radioisotopes in inducing DNA damage, activating DNA repair pathways, and their biological effects.

Radioisotopes	Emitting	Labeled	Mechanism of DNA Damage	DNA Repair Pathways	Biomarkers	Ref.
^223^Ra	α-emitter	-	DNA DSB and clustered DNA damage	NHEJ	❖ Up-regulation of 53BP1 and γ-H2AX	[52,53,54,58]
^212^Pb	α-emitter	-	DNA DSB	HR	❖ Down-regulation of Rad51 protein, inhibit Chk1 phosphorylation ❖ Cell cycle arrest (G2-M and S-phase) ❖ Alterations in protein levels associated with p73/GADD45 signaling pathway	[59,60,61]
		HER2				
		TCMC				
^213^Bi	α-emitter	E-cadherin	-	-	❖ Up-regulation of TNF, SPHK1, STAT5A, p21, MYT1, and SSTR3 mRNA ❖ Down-regulation of SPP1, CDC25 phosphatases mRNA	[62,63,64]
		CD20			❖ Activation of caspase 2, 3, 8, and 9 proteins ❖ Cell cycle arrest in G2/M-phase ❖ Downregulation of XIAP and Bcl-x proteins	
		CD45	Irreversible DNA DSB	NHEJ	❖ Activation of caspase 2, 3, 8, and 9 proteins ❖ PARP cleavage	
^125^I	AE	-	DNA DSB	-	-	[100,108,109]
		CEA	DNA DSB and ROS-mediated pathway	-	Enhanced formation of 53BP1 and γ-H2AX foci	
^111^In	AE	γH2AX	Lethal DNA DSB damage	-	Enhanced formation of ɤ-H2AX foci	[103,104,106,107]
		Anti-CD33				
		anti-HER2	Induced significant DNA DSBs			
^99m^Tc	AE	HYNIC-DAPI	Induced SSBs and DSBs via a direct interaction with DNA	-	-	[99]
^177^Lu	β-emitter	DOTATATE	Induction of indirect DNA damage through ROS generation and formation of SSBs	-	Slightly increased γH2AX and pATM	[137]
		DOTATATE and DOTATOC	time- and dose-dependent induction of DNA-DSBs		Enhanced formation of γ-H2AX and 53BP1 nuclear foci	[126,135,136]
		PSMA				
		HER2	DNA DSBs	NEHJ	❖ Activating caspase-3-mediated apoptosis ❖ Interfering with DNA-PK gene expression ❖ Downregulation of various genes involved in DDR, including BRCA1, EXO1, FEN1, MSH2, NBN, PRKDC, and RAD51	[138]
		DOTA-JR11, SSTR antagonist DOTA-octreotide, SSTR agonist	Reversable DNA DSBs	-	Enhanced the formation of 53BP1 and γ-H2AX foci	[132]
		EDTMP and DOTMP	-		❖ Downregulation of bcl-2 protein ❖ Cleavage of PARP protein, which serves as a substrate for active caspase-3 during cell death	[143]
		Minigastrin analog	-	Activation of DNA damage response by p53	-	[145]
^90^Y	β-emitter	-	Induction of indirect DNA damage through ROS generation and formation of SSBs	NEHJ	-	[68,123]
^131^I	β-emitter	-	Induction of indirect DNA damage through ROS	--	❖ Activation of initiator caspases-2, -8, -9, and effector caspase-3, along with the cleavage of PARP ❖ Down and up-regulating mRNA level of Bcl-2 and Fas, respectively ❖ Up-regulation of GADD45 mRNA, leading to G2/M phase arrest through a p53-independent pathway ❖ Variations in the expression of DNA repair genes, RAD51 and P21	[153,154]
^89^Sr	β-emitter	-	-	-	❖ Up-regulation of Fas acceptor and P53 mRNA ❖ Down-regulation of Bcl-2 mRNA	[141,151]

HR: homologous recombination, NHEJ: non-homologous end joining, SSB: single-strand break, DSB: double-strand break, β: beta, α: alpha, ^223^Ra: rhodium-223, AEs: Auger electrons, ^212^Bi: bismuth-212, ^125^I: iodine-125, ^111^In: indium-111, ^99m^Tc: technetium-99m, ^177^Lu: lutetium-177, ^131^I: iodine-131, ^90^Y: yttrium-90, ^89^Sr: strontium-89, γ-H2AX: gamma-H2AX, 53BP1: p53-binding protein 1, CEA: carcinoembryonic antigen, DAPI: 4,6-diamidino-2-phenylindole, HYNIC (6-hydrazinonicotinic), FasL: CD95 ligand, FasR: CD95 receptor, bcl-2: B-cell lymphoma 2, EDTMP: ethylenediamine tetramethylene phosphonic, GADD45: growth arrest and DNA damage-45, PARP: poly(ADP-ribose) polymerase.

## 3. The Role of DNA Damage Repair Pathways in Response to Radiopharmaceuticals

The DNA repair pathways are often compromised in cancer cells, rendering them more susceptible to the effects of radiotherapy and DNA-damaging agents. External beam and systemic RPT exploit these inherent vulnerabilities in the cancer cells by inducing DNA damage that exceeds their repair capacity, ultimately leading to cancer cell death [5,13]. To this end, two retrospective studies investigated DNA damage response gene mutations in mCRPC patients treated with ^223^RaCl_2_ therapy. These studies identified various DDR mutations, most frequently affecting genes such as ATM, BRCA2, ATR, CHEK2, FANCG, FANCI, PALB2, and CDK12. Patients lacking DDR ability showed significant improvements in overall survival and were more likely to complete ^223^RaCl_2_ therapy [155,156]. Conversely, the results of a recent retrospective study in mCRPC patients treated with ^223^RaCl_2_ indicated that mutations in tumor protein 53 (TP53), breast cancer genes1/2 (BRCA1/2), and phosphatase and tensin homolog (PTEN) are not reliable indicators of treatment response. There was no significant association between impaired DDR and overall or progression-free survival in these patients [157]. A prospective study revealed that DDR abnormalities were linked to higher membrane PSMA expression in PCa patients, potentially leading to a more favorable response to PSMA-targeted RPT [158]. Clinical outcomes of ^225^Ac-PSMA-617 therapy in two patients with mCRPC with DDR gene mutations, especially BRCA1, resulted in longer survival compared to patients without DDR mutations [159]. Additionally, a case study showed that a mutation in the BRCA2 gene, involved in the HR pathway, increased sensitivity to ^177^Lu-PSMA-617 therapy in patients with PCa [160]. An in vitro study demonstrated that reducing the expression of apurinic/apyrimidinic endonuclease 1/redox factor 1, a multifunctional protein pivotal in both DNA repair activity and reduction-oxidation activity, in human pancreatic cancer cells enhanced sensitivity to chromic-P32 phosphate, ^32^P-CP, therapy [161].

It is generally anticipated that RPT using α-emitters less commonly leads to resistance in cancer cells, likely owing to the induction of irreversible DSB as compared to the sticky-ended or SSBs caused by β-emitters [11,162]. However, even in response to α-emitters, multiple DDR mechanisms and signaling pathways in cancer cells can contribute to the development of resistance [163,164,165]. The selection of a repair pathway for DNA DSBs is a complex process influenced by multiple factors, including the quality [83] and the overall number of DSBs [162]. It has been observed that the resistance of leukemia cells to β-emitter, γ-irradiation, and doxorubicin is mediated by the NHEJ DNA repair mechanism [63]. Conversely, the induction of DNA damage by the α-emitter, ^213^Bi labeled with anti-CD45, was not effectively repaired by NHEJ and led to apoptosis [63]. Numerous studies have reported that mutations in DNA damage repair-associated genes can either increase or decrease the radiosensitivity of PCa [82,166,167,168]. In a study involving mCRPC patients treated with ^225^Ac-PSMA-617, various mutations in DDR and checkpoint genes such as TP53, CHEK2, ATM, BRCA1, BRCA2, PALB2, MSH2, MSH6, NBN, FANCB, and PMS1 were identified in the non-responders [169]. Furthermore, mutations in the TMPRSS2-ERG and retinoblastoma genes were found to confer resistance to ^223^RaCl_2_ therapy in mCRPC patients and were associated with worse OS [157].

## 4. Combination of Radiopharmaceuticals and DNA Damage Repair Inhibitors

Currently, the combination of inhibiting key proteins involved in the DDR with targeted RPT has emerged as a highly promising strategy to overcome the radio resistance of cancer cells (Figure 2B). The rationale underlying this strategy is to augment the efficacy of conventional treatments by targeting DNA damage response pathways, thus enhancing the overall response to treatment [170,171]. For instance, a combination of the α-emitter ^227^Th with mesothelin-targeted mAb (MSLN-TTC), along with ATR and PARPi showed superior anti-tumor effects in ovarian cancer xenograft models compared to MSLN-TTC alone. Additionally, these combinations resulted in reduced cell viability, as evidenced by the accumulation of higher levels of DSBs, indicated by increased γH2AX foci, and activation of apoptotic pathways [172]. Similar effectiveness was observed by combining ^227^Th-targeted with an anti-fibroblast growth factor receptor 2 (FGFR2-TTC) and ATRi, leading to increased levels of γH2AX and cell cycle arrest compared to using either treatment alone in various cancer cells [173]. Notably, this combination at a lower dose (100 kBq/kg) exhibited significant tumor growth inhibition in mouse xenograft models, whereas targeted α-therapy monotherapy had no significant therapeutic effect at the same dosage [173]. A synergistic effect was shown with the combination of a ^227^Th-targeted HER2 and PARPi, Olaparib, in a BRCA2 mutant human colorectal adenocarcinoma xenograft model [174]. Furthermore, the PARPi, MM4, functionalized with ^211^At demonstrated radiosensitivity in a neuroblastoma xenograft model, not only relying on enzymatic inhibition of PARP-1 to induce DNA damage but also amplifying the direct DNA DSBs caused by α-particle [175]. In another study, the combination of ^223^RaCl_2_ with DNA-PKi had varying impacts across different cancer cell lines. The addition of ATMi, on the other hand, showed the most promising results when combined with α-radiation, promoting the formation of micronuclei in cancer cells. However, the sensitization of cancer cells to X-ray radiation by DNA-PKi and ATMi varied depending on the specific characteristics of the cancer cells themselves [176]. Preliminary results from a phase I trial involving chemotherapy-naive patients with mCRPC demonstrated promising activity of the ^223^RaCl_2_ and PARPi, niraparib, regimen [177]. Additionally, combining an ATMi, KU59403, with the androgen receptor antagonist Enzalutamide (ENZA) and α-emitter, ^225^Ac-PSMA617, led to a higher level of apoptosis in non-responsive PCa cells. Researchers concluded that while ENZA enhanced radiosensitivity, concurrent activation of DDR necessitated the addition of an ATMi to improve the efficacy of treatment [178]. Recent findings revealed that the combination therapy of ^225^Ac-PP-F11N, a minigastrin analog, with a P53BP1 histone deacetylase inhibitor exhibited synergistic effects by enhancing cytotoxicity and inducing DNA damage in overexpressed cholecystokinin B receptor cancer cells, leading to improved treatment outcomes in preclinical models [179].

On the other hand, AEs offer the advantage of localized radiation delivery to the nucleus, which is the primary location of PARP-1. Radiolabeled PARPi have emerged, relying on radiation-induced DNA damage, in contrast to conventional PARPi that primarily function as enzymatic inhibitors. Both approaches leverage PARP-1’s role in DNA repair for therapeutic benefit but differ in their mode of action and the type of damage they induce [180,181]. For instance, the radioiodinated PARPi, ^125^I-KX1, generated AE in close proximity to DNA, resulting in the induction of DNA damage in both BRCA1 and non-BRCA mutant ovarian cancer cells. This leads to the accumulation of DNA damage and subsequent cell death [181]. It is noteworthy that ^125^I-KX1 was found to be twice as effective as the β-emitting ^131^I-KX1, in inducing an increase in DNA DSBs across a panel of neuroblastoma cell lines [180]. Additionally, the ^123^I-Meitner-Auger PARP1i showed prolonged survival in mice bearing glioblastoma compared to a vehicle control group, highlighting its potential therapeutic efficacy [182].

The combination therapy approach has demonstrated effectiveness in overcoming resistance to β-radiotherapy, particularly in glioblastomas that have exhibited resistance to such treatment. To address this challenge, the combination of ^131^I in either topoisomerase I, topotecan, or PARA, A-966492, inhibitors led to a significant increase in cell death and γ-H2AX foci in glioblastoma cells [183]. Furthermore, in a mouse model of glioblastoma, the administration of ^131^I-PARPi activated p53 expression and significantly prolonged the overall survival of the tumor-bearing mice [184]. In addition, it was found that PARPi led to enhanced cytotoxic effects of ^177^Lu-octreotate on both two-dimensional monolayer and three-dimensional spheroid models of neuroendocrine tumor cells expressing SSTR2 and SSTR5. This enhancement occurred by inducing cell cycle arrest and cell death processes [131]. Two other studies demonstrated a similar synergistic effect of ^177^Lu-DOTA-octreotate in combination with PARPi, talazoparib, and olaparib. These combinations resulted in increased DNA DSBs, as assessed by γ-H2AX foci formation, and significantly enhanced the in vivo anti-tumor efficacy [185,186]. Additionally, Fu et al. showed that the PARPi, fluzoparib, could potentiate the antitumor effect of ^177^Lu-DOTATATE in NCI-H727 cells synergistically. This effect was achieved by arresting the cell cycle in the G1 phase and reducing the tumor volume [187]. Furthermore, the combination therapy of ^177^Lu-DOTATATE with PARPi, Olaparib, reduced survival in different cell lines compared to RPT monotherapy [137]. However, it is important to note that despite the results in many studies, preclinical investigations showed that the combination of ^177^Lu-PSMA with different PARPi, including veliparib, olaparib, and talazoparib, did not result in a synergistic antitumor effect in PCa [188]. Furthermore, studies conducted by our group have shown that the combination of ^177^Lu-DOTATATE and ^90^Y radiolabeled granzyme B peptide, in combination with immune checkpoint inhibitor therapy, significantly improves treatment response in diverse animal cancer models compared to monotherapies [189,190].

## 5. Impact of Dose Rate on DNA Damage and Repair

In the realm of RPTs, dosimetry is less precisely defined compared to established protocols in EBRT. This lack of precise dosimetry complicates the estimation and comprehension of absorbed doses and their distribution within targeted tissues or organs during RPT [191,192]. The dose rate (DR) plays a pivotal role in determining the nature and magnitude of radiation-induced effects on cellular structures, gene expression, subsequent cellular responses, and the mode of cell death. Radiopharmaceuticals release radiation gradually over an extended period, with a continuously fluctuating and exponentially declining DR. The energy and distribution of dose as a function of diameter or depth differ for radionuclides. The DR in RPT is influenced by various factors, such as the radionuclide’s physical half-life, specific activity, and the biological half-life of the carrier (for example, antibody, ligand, or peptide), as well as the ability of cells to repair damage. In lower DR, the radiation is more dispersed and generally less harmful than in higher DR, which therefore results in reparable sublethal damage [191,193]. For example, administering a total accumulated dose 400 times higher than the natural background level at a low DR did not lead to significant increases in DNA damage. In contrast, when an equivalent dose was administered at high DRs, various forms of DNA damage, including base damage, micronuclei formation, and the expression of the p53 gene, became readily detectable. This implies that the rate at which the dose is delivered plays a critical role in determining the extent and nature of DNA damage observed in biological systems [194,195,196].

The evaluation of the impact of DR on DNA damage and cellular repair mechanisms entails assessing radiation-induced foci (RIF), which are represented by changes in the level of ɤ-H2AX and 53BP1 foci per cell. This method is utilized to determine the dose rate effectiveness factor (DREF) [195]. When cells were exposed to high DRs, a linear increase in ɤ-H2AX foci was observed. In contrast, delivering the same dose at low DRs resulted in a minimal increase in the ɤ-H2AX foci. This suggests that exposure at low DRs permits a substantial amount of induced damage to be repaired over the time required to deliver the dose, indicating a high DR effectiveness factor significantly greater than 1. This implies that high DRs are considerably more effective in causing DNA damage compared to low DRs [197,198].

However, some studies have suggested that the formation of the repair centers was not directly proportional to the radiation dose. Instead, they observed a non-linear increase in repair foci at low dose rates. This non-linear relationship might imply that the effectiveness of repair after exposure to low DRs could be more pronounced compared to high DRs [199]. These divergent findings contradict previous studies that suggested low doses of radiation did not activate genes necessary for DNA repair and demonstrated limited or no repair of DNA damage following low-dose exposures [200,201]. As both DNA repair and production of RIFs decline rapidly over time, their behavior concerning dose rate might be considerably lower and of limited utility in establishing a DREF. Additionally, it was shown that the production of micronuclei (MN) in human B lymphoblast cells exposed to ^241^Am and ^137^Cs, a γ-ray emitter, was influenced by the DR. The findings revealed that the relationship between the dose and induction of MN caused by γ-rays corresponded well to the linear-quadratic model. Conversely, the induction of MN due to α-emitter irradiation displayed a biphasic pattern, indicating hypersensitivity at low doses [202]. Sebastien et al. conducted a comprehensive comparative study on the repair of radiation-induced DNA damage ex vivo in 15 strains of mice subjected to both low- and high-LET radiation. The results showed that initially, there was a higher saturation of RIF per dose at 4 h post-irradiation, showing increased RIF/gray (Gy) for lower LET radiation (X-rays and ^40^Ar) in comparison to lower LET. However, at later time intervals (24 h and beyond), there was a reversal in this pattern, displaying a higher RIF/Gy for higher LET radiation, suggesting that the probability of encountering a greater number of DSBs per RIF also increases. Consequently, this hinders cells from fully resolving RIF caused by high-LET radiation, elucidating the heightened sensitivity to high-LET radiation despite a lower number of RIF being observed [198].

In a study by Manning et al., human blood was exposed to high or low doses of low-LET ionizing radiation. The dose-response relationship following high doses of radiation was described by a polynomial expression of the p53 gene, indicating a more complex relationship between dose and gene expression. Conversely, the dose-response relationship following low doses was linear, suggesting a straightforward correlation between dose and gene expression at lower exposure levels [203]. Another study by Ghandhi et al. suggested that some of the p53-regulated genes responded to radiation exposure, regardless of whether it was a high or low dose delivered at either low or high DRs. This implies that certain genes regulated by p53 were sensitive to radiation exposure across different dose ranges and DRs, potentially highlighting their importance in the cellular response to radiation-induced DNA damage [204]. The response to acute exposure to cobalt-60 (^60^Co) in studies conducted in vitro has demonstrated a non-linear relationship between dose and biological effect, implying that a single value for a DREF cannot be derived. At higher doses of low-LET radiation delivered at a high DR, there is a higher occurrence of chromosome aberrations compared to the same dose delivered at a low DR. Therefore, at higher doses, estimating a consistent DREF becomes challenging since it varies with the received dose [205]. Various studies have suggested that DNA damage repair in low DR is limited. This limitation was hypothesized to be due to the insufficient induction of DNA damage at low doses, which fails to trigger the adequate expression of DNA repair genes. These findings indicate that the cellular response to DSBs varies significantly between low and high doses of low-LET radiation [200,206,207,208,209]. The disparity in cellular response implies that if DNA repair mechanisms are not active at low doses and if the extent of DNA damage directly correlates with cancer risk, then the risk of cancer should increase linearly with the dose. Moreover, the absence of repair mechanisms at low doses could potentially result in a higher cancer risk than that extrapolated linearly from high doses [196,201].

The bystander effect is another indirect damage mechanism induced following exposure to RPT [210,211]. It refers to the transmission of damage from irradiated cells to neighboring non-irradiated cells [33,212]. In numerous in vitro studies, tritium (^3^H), a β-emitter, has been used to label nucleobases such as deoxythymidine (^3^HTdR) or deoxycytidine (^3^HdC). These labeled nucleobases are generally confined within the cell nucleus, thus avoiding the irradiation of neighboring cells. However, the incorporation of ^3^HTdR has shown detrimental effects on non-irradiated cells, including inhibiting cell proliferation, suppressing clonogenicity, prompting cell death, causing chromosome aberrations, inducing DNA strand breaks, and leading to cell cycle arrest [213,214,215,216]. Bystander mutagenesis induced by radionuclides has been observed in spheroid cells labeled with tritiated thymidine ([^3^H] dTTP), resulting in a significant 14-fold increase in mutations and a decrease in clonogenic survival within the non-irradiated cells [35,217]. Studies conducted on 3D cell models have demonstrated that ^125^IUdR led to an increase in lethal bystander effects [218,219]. Sedelnikova et al. discovered multiple damages, including DNA DSBs visualized as ɤ-H2AX foci, generation of MN, apoptosis, senescence, and alterations in DNA methylation in bystander cells following microbeam irradiation of 3D artificial tissues such as skin or respiratory epithelium [220]. Additionally, it was reported that the DNA damage, measured by sister chromatid exchanges, occurred not solely due to the direct effect of ^238^Pu, an α-emitter, through the cell nucleus but also due to the generation of ROS factors, which are believed to be the initiators of the bystander effect [211]. Fu et al. noted a bilateral interaction between human bronchial epithelial cells (Beas-2B) irradiated with α-particles and their bystander macrophage U937 cells. Specifically, they observed that when Beas-2B cells were irradiated with ^241^Am, α-emitter, it resulted in a significant increase in apoptosis and a decrease in survival in the bystander U937 cells [221]. A protective or rescue bystander effect was discovered in cancerous HeLa cells when they were exposed to very low doses of ^241^Am and co-cultured with NIH/3T3 fibroblasts. This effect, characterized by a decrease in 53BP1 foci per cell, was driven by the activation of the nuclear factor kappa B (NF-kB) pathway within the irradiated cells themselves. These findings highlight the complexity of the bystander effect and imply that cell phenotype characteristics could contribute to observed variations. Additionally, the signaling observed between tumor cells and normal cells could significantly impact the therapeutic outcomes of cancer radiotherapy involving radionuclides, particularly given the coexistence of cancer cells alongside normal cells such as fibroblasts or others [222]. Boyd et al. conducted a comparison between the induction of the bystander effect by ɤ-rays and halo-analogs of metaiodobenzylguanidine (MIBG) radiolabeled by isotopes with varying LET, including ^131^I-MIBG (low-LET β-emitter), ^123^I-MIBG (potentially high-LET AE emitter), and ^211^At-MABG (high-LET α-emitter), to expose two human tumor cell lines. In their experiments, non-irradiated cells were exposed to the medium collected from cells that had accumulated the radiopharmaceuticals or were directly irradiated with external ɤ-rays. They found that ɤ-irradiation induced a bystander effect, measured as a reduction in clonogenic survival, which increased with the dose delivered to donor cells at lower doses and saturated afterward. Conversely, the low-LET ^131^I-MIBG-induced bystander cell death increased with the dose delivered to donors and did not reach saturation levels even after treatment with a range of radioactivity causing direct cell death comparable to external ɤ-irradiation. However, high-LET emitters, specifically ^123^I-MIBG and ^211^At-MABG, induced increased killing of recipient cells to levels similar to direct kill, approximately ~65% and 70%, respectively. Subsequently, the effect on recipients decreased with increasing activities among donors, resulting in U-shaped bystander curves [223]. They also demonstrated that ^131^I-MIBG or ^131^I-UdR induced bystander cell killing in colorectal carcinoma cells. This effect increased with the dose and did not reach saturation levels even after treatment with higher doses. In contrast, high-LET emitters, specifically ^123^I-labeled compounds, induced U-shaped curves of bystander cell killing [37]. This effect suggests that lower damage could have been induced by higher dose-rate ^123^I-labeled cells compared to the same dose delivered at a lower dose rate with ^131^I-labeled cells. Consequently, it is plausible that bystander signaling was generated less efficiently at the higher dose rate than at the lower dose rate. Similarly, in vitro co-culture experiments involving human colon cancer cells exhibited varied bystander effects: Inhibitory effects were observed with ^125^I, while stimulatory effects were noticed with ^123^I [224]. Xue et al. emphasized a substantial inhibitory bystander effect on tumor growth observed in a mouse model using lethal doses of DNA-incorporated ^125^IUdR. This effect was mediated by factors generated within the ^125^IUdR-labeled cells and subsequently secreted, playing a pivotal role in suppressing tumor growth [89,225]. While Kishikawa et al. [72] observed contrasting effects in human adenocarcinoma cells damaged by different AEs, specifically ^123^I (^123^IUdR) and ^125^I. Upon injecting these damaged cells subcutaneously into nude mice as a mixture with unlabeled cells, they discovered that ^123^I-labeled cells significantly enhanced tumor growth, whereas ^125^I-labeled cells notably inhibited tumor growth. Despite both isotopes emitting AEs, they differ in their physical half-lives, resulting in varying dose rates; ^123^I-labeled cells exhibit a rate 109 times higher than that of ^125^I-labeled cells. Additionally, the number of decays per cell after in vivo incubation with ^125^IUdR is approximately twice that calculated for ^123^IUdR. These findings strongly indicate the necessity of evaluating bystander effects for different radionuclides at various doses before drawing conclusions regarding the bystander effect phenomenon [226].

In a study using ^211^At to examine the influence of radiation-induced biological effects on thyroid tissue in BALB/c nude mice, distinct gene expression profiles were observed in the thyroid tissue exposed to various absorbed doses compared to non-irradiated controls. Interestingly, a greater number of genes were affected at low-absorbed doses in comparison to intermediate and higher-absorbed doses. These affected genes were found to be involved in critical cellular functions such as metabolism, transport and communication, DNA, RNA, and protein processing, immune response, apoptosis, cellular maintenance, and cell development. Additionally, it was noted that downregulation of genes was more prevalent at lower absorbed doses, while upregulation was more pronounced at higher absorbed doses. As irradiation at low absorbed doses induced changes in a larger number of gene expressions, the authors suggest that this inverse response partly originates from non-irradiated bystander cells within the tissue [226]. Furthermore, Mitrofanova et al. documented a substantial growth inhibition of human prostate xenografts induced by DU145 cells that were transduced with the human sodium iodide symporter (NIS) gene. Interestingly, a low dose of ^131^I-NaI effectively hindered the growth of relatively large tumors. Given that not all cells were efficiently transduced with the NIS gene, the observed inhibition of tumor growth was inferred to occur through bystander effects [227].

## 6. Knowledge Gap

To address the knowledge gap regarding the influence of various radioisotopes on DNA damage and repair pathways, it is imperative for researchers to undertake comprehensive comparative studies. Understanding how distinct radioisotopes impact DNA damage and repair mechanisms, dosimetry-based estimation of the absorbed dose, and head-to-head comparison of the effects of radioisotopes with different energy and emission ranges and the impact of photonics compared to electronic emission components of the radioisotopes is pivotal for advancing the field of RPT. In addition, more efforts into improving the preclinical models, exploring the tumor impact of RPTs on the tumor microenvironment, further investigating the combination treatment strategies, and thorough evaluation of the short- and long-term toxicity of the RPTs are required. Bridging this gap necessitates interdisciplinary collaboration among oncologists, nuclear medicine physicians, radiation oncologists, radiation physicists, biologists, and geneticists. Leveraging advanced genomic and proteomic techniques can yield insights into cellular responses to radiation stemming from different radioisotopes. Furthermore, exploring the long-term effects of radioisotope-induced DNA damage and repair pathways can offer valuable insights into health risks and contribute to the development of radiation safety guidelines, thus ensuring continued engagement from the scientific community in closing this knowledge gap.

## 7. Conclusions

Here we provide a comprehensive overview of the scientific literature exploring the role of different radioisotopes in causing direct and indirect DNA damage as well as their impact on the activation of DNA repair pathways in cancers. The existing evidence suggests that high-energy α-emitter radioisotopes can directly interact with the DNA molecule, leading to ionization and the subsequent formation of ionized atoms or molecules. This ionization process primarily results in irreparable and complex DSBs. Conversely, most of the DNA damage induced by β-emitter radioisotopes occurs indirectly through the generation of free radicals, such as ROS, and subsequent chemical reactions. Beta-particles themselves can also collide with the DNA molecule, leading to SSBs and potentially reversible DSBs. The induction of direct or indirect DNA damage resulting from the AE depends on various factors, including the energy of the AE, its proximity to the DNA molecule, and the surrounding cellular environment. Within cancer cells, multiple DNA repair mechanisms and signaling pathways can contribute to the development of resistance in response to RPTs. Currently, a promising strategy for overcoming resistance involves combining therapy with agents that inhibit key proteins involved in the DDR alongside RPT. This approach holds substantial potential for enhancing treatment efficacy and addressing the challenges posed by radioresistant cancer cells.

## Figures and Tables

**Table 1:** Distinct physical and biological properties of alpha, beta, and Auger electron emitting radioisotopes [17,30,31,33,34,35,36,37,38].

	Alpha Particle	Beta Particle	Auger Electron
Type of particles	^4^He nuclei	Energetic electrons	Low-energy electrons; electron capture and/or internal conversion
Energy range	4–9 MeV	50–2300 KeV	25–80 KeV
Emission range in tissues	28–100 µm	0.5–10 mm	<0.5 µm
LET (KeV/µm)	~50–230	~0.1–1.0	~4–26
Main mechanism of damage	At high doses: widespread DNA damage, leads to significant cellular damage and reduced repair capability to induce cell death or mutations with potential long-term effects. At low to moderate doses: DSBs, less reparable by cellular mechanisms.	At high doses: exponential relationship with tumor survival. The rate of DNA damage may exceed the cell’s repair capacity, leading to the accumulation of unrepaired or misrepaired DNA lesions. At low to moderate doses: linear relationship with tumor survival. Primarily involves SSBs and minor chemical modifications to DNA bases. Damage is more likely to be repaired by the cell’s repair mechanisms.	At high doses: multiple DSBs, lead to increased genetic instability and potential cell death. At low to moderate doses: clustered DNA damage, leads to complex lesions that overwhelm repair systems.
Tissue damage size	Small (a number of cells)	Higher volume solid tumor	Micro (a few cells)

LET: linear energy transfer, SSB: single-strand break, DSB: double-strand break, mm: millimeter.

## Data Availability

Data are available on the list of referenced articles.

## Supplementary Materials

The following supporting information can be downloaded at: https://www.mdpi.com/article/10.3390/pharmaceutics15122761/s1, Table S1. Characteristics of radionuclides used in radiopharmaceutical therapy.
