# Supplementary material for: DNA Damage by Radiopharmaceuticals and Mechanisms of Cellular Repair
Source: Pharmaceutics. 2023 Dec 12;15(12):2761. doi: 10.3390/pharmaceutics15122761 (PMC10748326; doi:10.3390/pharmaceutics15122761)
Supplement: Supplementary file 1 [file pharmaceutics-15-02761-s001.zip › pharmaceutics-2738033-supplementary.pdf]

Table S1. Characteristics of radionuclides used in radiopharmaceutical therapy.

Radionuclides	Particle Emission	Half-Life	Particle Energy (mean MeV)	Maximum Emission Range in Tissue (mm)	Ref
^{225}**Ac**	α/EC	10 d	0.21	0.061	[1-5]
^{213}**Bi**	α/β−	46 mins	8.32	0.084	[6-8]
^{211}**At**	α	7.2 h	6.79	0.067	[1,7,8]
^{223}**Ra**	α/γ	11.44 d	6.59	0.08	[1,4,9,10]
^{177}**Lu**	β−/γ	6.73 d	0.208	1.6	[5,7-12]
^{153}**Sm**	β−/γ	46.50 h	0.808	4	[1,2,8-11]
^{90}**Y**	β−	2.67 d	1.35	12	[6-12]
^{89}**Sr**	β−/γ	50.53 d	0.908	7	[2,8-11]
^{131}**I**	β−/γ	8.02 d	0.356	2.3	[2,8-13]
^{3}**H**	β−	12.32 y	0.018	-	[1,2,6-9]
^{32}**P**	β−/γ	14.26 d	0.695	8.0	[1,2,8]
^{137}**Cs**	γ/β−	30 y	0.5120	-	[1,2,6-8]
^{60}**Co**	γ/β−	5.27 y	1.1732	-	[1,2,3]
^{123}**I**	γ/EC	13 h	0.013	0.001	[3,5,12-15]

^{125}I	γ/AE	57 d	0.023	0.0001	[3,12,16,17]
^{111}In	γ/EC	67 h	0.007	0.04	[3,18-22]
$^{Tc-99}$m	γ/AE	6 h	0.005	-	[3,23]

EC: Electron Capture, AE: Auger Electron, d: Day, h: Hour, y: Year, mins: Minutes.

References

1. Yeong, C.-H.; Cheng, M.-h.; Ng, K.-H. Therapeutic radionuclides in nuclear medicine: current and future prospects. *Journal of Zhejiang University. Science. B* **2014**, *15*, 845.
2. Elliyanti, A. Radiopharmaceuticals in modern cancer therapy. In *Radiopharmaceuticals-Current Research for Better Diagnosis and Therapy*; IntechOpen: 2021.
3. Ku, A.; Facca, V.J.; Cai, Z.; Reilly, R.M. Auger electrons for cancer therapy–a review. *EJNMMI radiopharmacy and chemistry* **2019**, *4*, 1-36.
4. Navalkissoor, S.; Grossman, A. Targeted alpha particle therapy for neuroendocrine tumours: the next generation of peptide receptor radionuclide therapy. *Neuroendocrinology* **2019**, *108*, 256-264.
5. Parsi, M.; Desai, M.H.; Desai, D.; Singhal, S.; Khandwala, P.M.; Potdar, R.R. PSMA: A game changer in the diagnosis and treatment of advanced prostate cancer. *Medical Oncology* **2021**, *38*, 1-20.
6. Salih, S.; Alkatheeri, A.; Alomaim, W.; Elliyanti, A. Radiopharmaceutical treatments for cancer therapy, radionuclides characteristics, applications, and challenges. *Molecules* **2022**, *27*, 5231.
7. Widel, M.; Przybyszewski, W.M.; Cieslar-Pobuda, A.; Saenko, Y.V.; Rzeszowska-Wolny, J. Bystander normal human fibroblasts reduce damage response in radiation targeted cancer cells through intercellular ROS level modulation. *Mutation Research/Fundamental and Molecular Mechanisms of Mutagenesis* **2012**, *731*, 117-124.
8. Elliyanti, A. Molecular radiobiology and radionuclides therapy concepts. *The Evolutionof Radionanotargeting towards Clinical Precission Oncology: A Festschrift in Honor of Kalevi Kairemo* **2022**, 395-408.
9. Hillegonds, D.J.; Franklin, S.; Shelton, D.K.; Vijayakumar, S.; Vijayakumar, V. The management of painful bone metastases with an emphasis on radionuclide therapy. *Journal of the National Medical Association* **2007**, *99*, 785.
10. Qaim, S.M. Therapeutic radionuclides and nuclear data. *Radiochimica Acta* **2001**, *89*, 297-304.
11. Asadian, S.; Mirzaei, H.; Kalantari, B.A.; Davarpanah, M.R.; Mohamadi, M.; Shpichka, A.; Nasehi, L.; Es, H.A.; Timashev, P.; Najimi, M. β-radiating radionuclides in cancer treatment, novel insight into promising approach. *Pharmacological research* **2020**, *160*, 105070.
12. Luster, M.; Pfestroff, A.; Hänscheid, H.; Verburg, F.A. Radioiodine therapy. In Proceedings of the Seminars in nuclear medicine, 2017; pp. 126-134.
13. Slonimsky, E.; Tulchinsky, M. Radiotheragnostics paradigm for radioactive iodine (Iodide) management of differentiated thyroid cancer. *Current Pharmaceutical Design* **2020**, *26*, 3812-3827.
14. Balagurumoorthy, P.; Wang, K.; Adelstein, S.J.; Kassis, A.I. DNA double-strand breaks induced by decay of 123I-labeled Hoechst 33342: role of DNA topology. *International journal of radiation biology* **2008**, *84*, 976-983.
15. Reske, S.N.; Deisenhofer, S.; Glatting, G.; Zlatopolskiy, B.D.; Morgenroth, A.; Vogg, A.T.; Buck, A.K.; Friesen, C. 123I-ITdU–mediated nanoirradiation of DNA efficiently induces cell kill in HL60 leukemia cells and in doxorubicin-, β-, or γ-radiation–resistant cell lines. *Journal of Nuclear Medicine* **2007**, *48*, 1000-1007.
16. Balagurumoorthy, P.; Xu, X.; Wang, K.; Adelstein, S.J.; Kassis, A.I. Effect of distance between decaying 125I and DNA on Auger-electron induced double-strand break yield. *International journal of radiation biology* **2012**, *88*, 998-1008.
17. Paillas, S.; Boudousq, V.; Piron, B.; Kersual, N.; Bardiès, M.; Chouin, N.; Bascoul-Mollevi, C.; Arnaud, F.-X.; Pèlegrin, A.; Navarro-Teulon, I. Apoptosis and p53 are not involved in the anti-tumor efficacy of 125I-labeled monoclonal antibodies targeting the cell membrane. *Nuclear medicine and Biology* **2013**, *40*, 471-480.
18. Nordberg, E.; Orlova, A.; Friedman, M.; Tolmachev, V.; Ståhl, S.; Nilsson, F.Y.; Glimelius, B.; Carlsson, J. In vivo and in vitro uptake of 111In, delivered with the affibody molecule (ZEGFR: 955) 2, in EGFR expressing tumour cells. *Oncology reports* **2008**, *19*, 853-857.
19. Costantini, D.L.; Chan, C.; Cai, Z.; Vallis, K.A.; Reilly, R.M. 111In-labeled trastuzumab (Herceptin) modified with nuclear localization sequences (NLS): an Auger electron-emitting radiotherapeutic agent for HER2/neu-amplified breast cancer. *Journal of Nuclear Medicine* **2007**, *48*, 1357-1368.
20. Cai, Z.; Chen, Z.; Bailey, K.E.; Scollard, D.A.; Reilly, R.M.; Vallis, K.A. Relationship between induction of phosphorylated H2AX and survival in breast cancer cells exposed to 111In-DTPA-hEGF. *Journal of Nuclear Medicine* **2008**, *49*, 1353-1361.
21. Chen, P.; Wang, J.; Hope, K.; Jin, L.; Dick, J.; Cameron, R.; Brandwein, J.; Minden, M.; Reilly, R.M. Nuclear localizing sequences promote nuclear translocation and enhance the radiotoxicity of the anti-CD33 monoclonal antibody HuM195 labeled with 111In in human myeloid leukemia cells. *J Nucl Med* **2006**, *47*, 827-836.
22. Chen, P.; Wang, J.; Hope, K.; Jin, L.; Dick, J.; Cameron, R.; Brandwein, J.; Minden, M.; Reilly, R.M. Nuclear localizing sequences promote nuclear translocation and enhance the radiotoxicity of the anti-CD33 monoclonal antibody HuM195 labeled with 111In in human myeloid leukemia cells. *Journal of Nuclear Medicine* **2006**, *47*, 827-836.
23. Kotzerke, J.; Punzet, R.; Runge, R.; Ferl, S.; Oehme, L.; Wunderlich, G.; Freudenberg, R. 99mTc-labeled HYNIC-DAPI causes plasmid DNA damage with high efficiency. *PLoS One* **2014**, *9*, e104653.
